# An AI-based auxiliary empirical antibiotic therapy model for children with bacterial pneumonia using low-dose chest CT images

**DOI:** 10.1007/s11604-021-01136-2

**Published:** 2021-06-08

**Authors:** Mudan Zhang, Siwei Yu, Xuntao Yin, Xianchun Zeng, Xinfeng Liu, ZhiYan Shen, Xiaoyong Zhang, Chencui Huang, Rongpin Wang

**Affiliations:** 1 Guizhou University, School of Medicine, Guiyang, 550000 Guizhou Province China; 2 Department of Radiology, Guizhou Provincial People’s Hospital, Guiyang, 550002 Guizhou Province China; 3 Guizhou Provincial Key Laboratory of Intelligent Medical Image Analysis and Precision Diagnosis, Guizhou Provincial People’s Hospital, Guiyang, 550002 Guizhou Province China; 4 AI Lab, Deepwise & League of PhD Technology Co. LTD, Beijing, China; 5 School of Clinical Medicine, Guizhou Medical University, No. 9 Beijing Road, Yunyan District, Guiyang, 550004 Guizhou Province China; 6 Smart Hospital Construction Office, Guizhou Provincial People’s Hospital, No. 83 Zhongshan East Road, Nanming District, Guiyang, 550002 Guizhou Province China

**Keywords:** Bacterial pneumonia, Radiomics, Children, CT, Multi-class classification

## Abstract

**Purpose:**

To construct an auxiliary empirical antibiotic therapy (EAT) multi-class classification model for children with bacterial pneumonia using radiomics features based on artificial intelligence and low-dose chest CT images.

**Materials and methods:**

Data were retrospectively collected from children with pathogen-confirmed bacterial pneumonia including Gram-positive bacterial pneumonia (122/389, 31%), Gram-negative bacterial pneumonia (159/389, 41%) and atypical bacterial pneumonia (108/389, 28%) from January 1 to June 30, 2019. Nine machine-learning models were separately evaluated based on radiomics features extracted from CT images; three optimal submodels were constructed and integrated to form a multi-class classification model.

**Results:**

We selected five features to develop three radiomics submodels: a Gram-positive model, a Gram-negative model and an atypical model. The comprehensive radiomics model using support vector machine method yielded an average area under the curve (AUC) of 0.75 [95% confidence interval (CI), 0.65–0.83] and accuracy (ACC) of 0.58 [sensitivity (SEN), 0.57; specificity (SPE), 0.78] in the training set, and an average AUC of 0.73 (95% CI 0.61–0.79) and ACC of 0.54 (SEN, 0.52; SPE, 0.75) in the test set.

**Conclusion:**

This auxiliary EAT radiomics multi-class classification model was deserved to be researched in differential diagnosing bacterial pneumonias in children.

## Introduction

Community-acquired pneumonia (CAP) is a leading cause of childhood morbidity and mortality worldwide. In 2015, approximately 700,000 children < 5 years of age died from CAP. The most common pathogens of CAP are viruses, followed by typical bacteria and then atypical bacteria [1]. According to 2016 Global Burden of Disease (GBD) data [2], approximately 64% of pneumonia deaths in children under 5 years old were bacterial in etiology.

Potentially life-threatening bacterial infections require immediate and precise antibiotic therapy. Because of the spread of antibiotic resistance, the altered microbial-community structure caused by antibiotic use and the fewer new antibacterials released in recent years due to the high costs involved, rational prescription of antibiotics for CAP is more important [3]. In practical clinical situations, it is very difficult to precisely prescribe antibiotics to individual children with CAP based on bacterial pathogens that are proven by testing to be the cause of infection. Reasons include the low yield of blood cultures, lack of satisfactory sputum specimens and unwillingness to perform pulmonary aspiration. Furthermore, positive results are scarce due to the appearance of resistant bacterial strains [4]. Therefore, most early-stage antibiotic therapy for bacterial pneumonia is empirical; that is, it covers a range of possible target bacteria while culture results are pending.

Currently, empirical antibiotic therapy (EAT) relies on confirmed data from relevant examinations of similarly infected patients that reflect the epidemiology of most types of bacterial infection, as well as distribution and risk factors. At present, such data mostly show antimicrobial-susceptibility testing results by bacterial species [5]. However, not all data may be of clinical relevance that should be verified more accurately and broadly. In addition, the physician might feel that antibiotics covering a very broad range of bacterial infections always work, although even the broadest-spectrum antibiotics have gaps in their coverage. In this case, the physician would try to use ever-more-complex combinations of antibiotics to ensure coverage of more possible bacterial infections. However, using more broad-spectrum options for children would increase antibiotic resistance locally and globally [3], and prolonged empirical use of broad-spectrum antibiotics or frequent changes of antibiotics are associated with a number of negative pathological-test outcomes [6]. Owing to the lack of practical guidance and evidence, some pediatricians even empirically choose antibiotics based on patients’ symptoms and signs and on their own subjective experience [7].

It would be more suitable to try to find an evidence-based way to assist EAT and then to focus on individual children. Currently, in the field of radiology, artificial intelligence (AI) and radiomics are viable, good-performing tools to support clinical decision making in diagnosis, prognosis and evaluation of lung diseases [8]. Eui Jin Hwang et al. [9] developed a deep-learning-based algorithm that can classify normal and abnormal results from chest radiographs with pneumonia and other major thoracic diseases; they noted that the algorithm demonstrated significantly higher performance than all three physician groups in their study. EAT is a growing field of AI application research in radiology, but until now there have been few research results, especially in children. In 2012, the application of an artificial neural network (ANN) to EAT for lower respiratory tract infections in the elderly was reported; the network predicted the resolution index 91.67% of the time [10].

Therefore, in our study, we hypothesized that radiomics can be used in differential diagnosis the types of pneumonias and we developed an AI-based radiomics multi-class classification model to assist EAT for children by classifying Gram-positive bacterial pneumonia, Gram-negative bacterial pneumonia and atypical pneumonia, including *Chlamydia pneumoniae* pneumonia (CPP), *Mycoplasma pneumoniae* pneumonia (MPP) and *Chlamydia trachomatis* pneumonia (CTP), using low-dose chest computed-tomography (CT) images. This model could offer objective evidence or recommendations for EAT in the future.

## Materials and methods

### Ethical approval and data collection

**Fig. 1:**
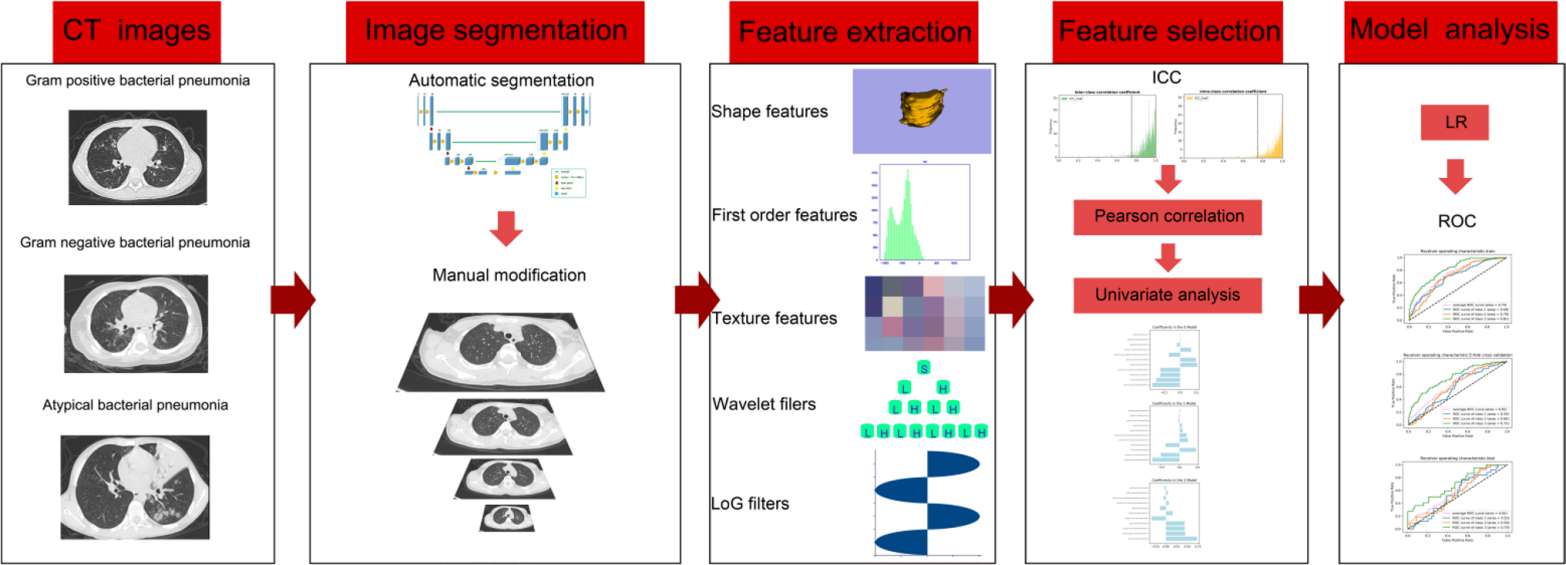
The workflow of the construction of radiomics model

Trained researchers extracted and recorded clinical data and CT images of the study population from hospital case notes and Picture Archiving and Communication System (PACS) at Guizhou Provincial People's Hospital, China. This study was approved by the Medical Ethics Committee of Guizhou Provincial People's Hospital. Because of its retrospective nature, the need to obtain informed consent from the patients was waived. We performed the study according to the principles of the Declaration of Helsinki. Figure 1 shows the work flow of the construction of the radiomics model.

### Inclusion and exclusion criteria

In this study, we included a total of 861 children < 14 years of age with CAP who were consecutively admitted to Guizhou Provincial People's Hospital from January 1 to June 30, 2019. They had clinical diagnoses and radiological evidence of CAP and clear etiological diagnoses. Gram-positive or Gram-negative bacterial etiology was tested from sputum cultures. We extracted atypical bacteria, including *M. pneumoniae*, *C. pneumoniae* and *C. trachomatis*, from nasopharyngeal specimens after detecting them using a quantitative diagnostic kit by Chemiluminescent Immunoassay produced by Guizhou Provincial People's Hospital Co., Ltd. Cases with no low-dose chest CT images (*n* = 404) or with bacterial coinfection (*n* = 68) were excluded.

**Fig. 2:**
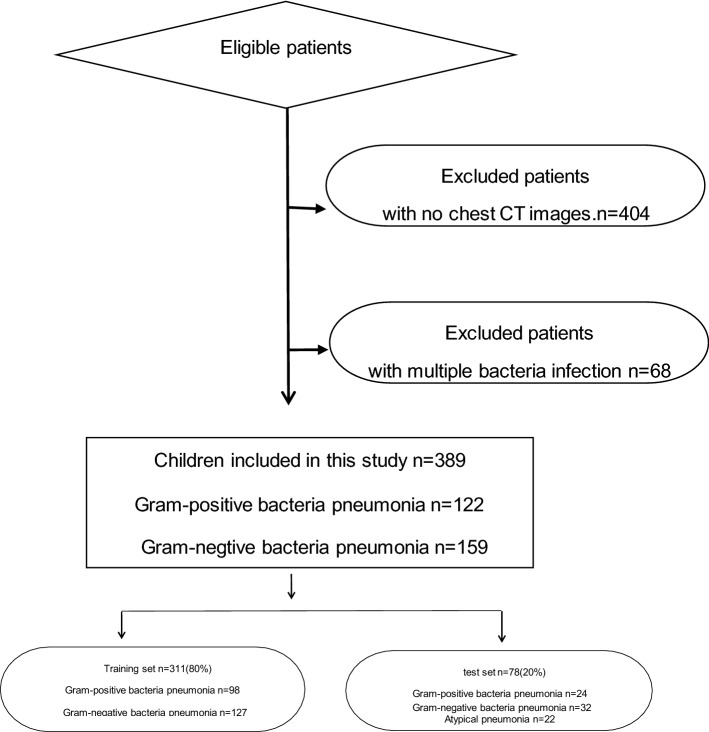
The inclusion and exclusion criteria

All patient data were randomly divided into two individual sets, a training set and a test set, at a ratio of 8:2. Figure 2 shows the inclusion and exclusion criteria.

### Chest CT characteristics

All children received non-enhanced chest CT at our hospital, covering the entirety of both lungs. CT images were obtained using a Siemens SOMATOM Force CT Scanner (Siemens Healthcare, Forchheim, Germany). Image parameters were as follows: 100 kV; 60 mAs; rotation time, 0.25 s; detector collimation, 2 × 192 × 0.6; field of view (FOV), 300 × 300 mm. All CT images were reconstructed into sections 1.00 mm thick. The radiation dose of a low-dose chest CT was within 1.0 mSv.

### Image processing

**Fig. 3:**
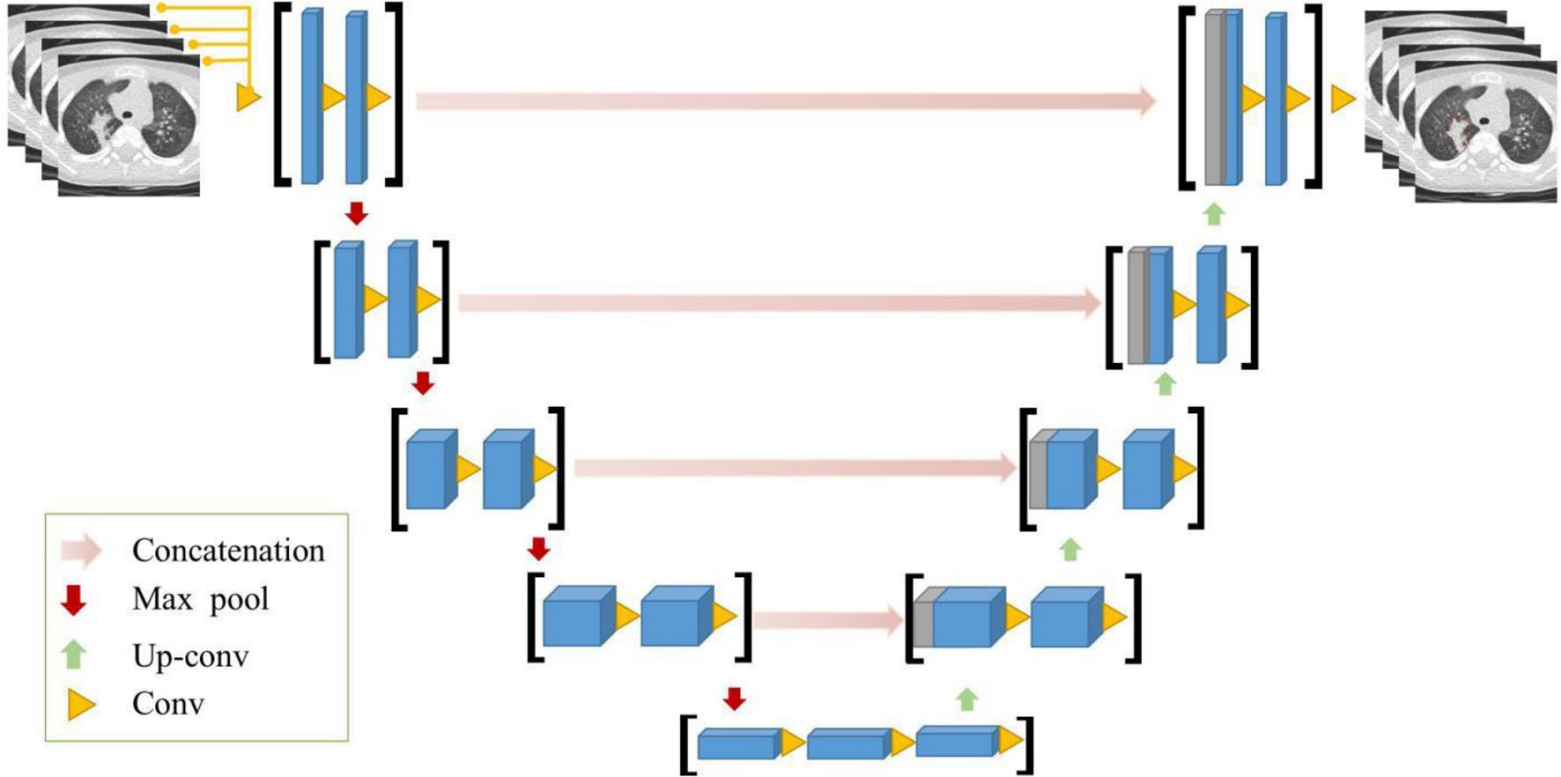
The architecture of automated segmentation model

First, we segmented region-of-interest (ROI) volumes using an automated segmentation architecture based on three deep-learning algorithms. The automated segmentation model is established by a U-Net, as the basic architecture for infection segmentation, which adopts pseudo-3D convolution as the building module. The input of the network is multiple CT slices, forming the 3D input and the 2D convolution layers were replaced by the 3D ones. All cross-sectional images were reconstructed by CT per case were used. Eight successive slices were taken into the segmentation model, and the segmentation results of eight slices were obtained. Then next eight non-overlapping slices entered into the model as new inputs and all the cross-sectional images were traversed. Finally, all the segmentation results of a CT case are obtained (Fig. 3). Evaluation of auto-segmentation accuracy was completed before image segmentation. Next, all ROIs were manually modified by a radiologist with > 5 years’ clinical experience and validated by a second radiologist with 10 years’ clinical experience. B-spline interpolation resampling was used to normalize voxel size, and anisotropic voxels were resampled to form isotropic voxels of 1.0 mm × 1.0 mm × 1.0 mm.

### Radiomics feature extraction and selection

We extracted radiomics features using Pyradiomics software 3.0 (https://pyradiomics.readthedocs.io/en/latest/; accessed July 6, 2019). Six common feature groups were extracted from low-dose chest CT images: first-order features, shape features, gray-level co-occurrence matrix (GLCM), gray-level run length matrix (GLRLM), gray-level size zone matrix (GLSZM) and gray-level dependence matrix (GLDM). Next, we standardized the training set using the standard scaler package in scikit-learn version 0.23.2 (https://scikit-learn.org/stable/modules/preprocessing.html) and applied the standardized model in the training set to the test set.

**Fig. 4:**
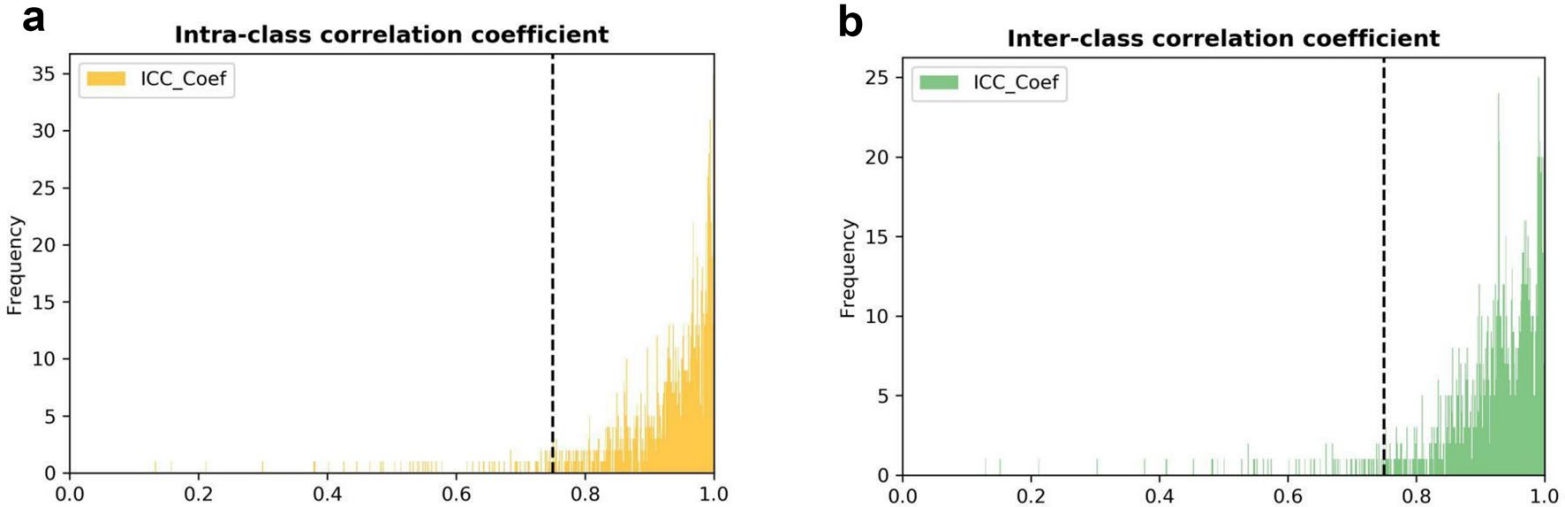
Histogram of the intra-class correlation coefficient and inter-class correlation coefficient. After robustness test, **a** 1154 and **b** 1152 of the initial 1218 CT images features were attained

Then, we performed a process of feature dimension reduction in which high-dimensional features were extracted to select the most relevant features. In addition, we used intra-class and inter-class correlation coefficients to evaluate the consistency of measurements made, respectively, by different observers measuring the same quantity and by the same observer measuring different quantities (Fig. 4). Features with an intra-class correlation coefficient > 0.75 and an inter-class correlation coefficient > 0.75 were considered to have satisfactory agreement and were selected for further analysis.

After the feature consistency test, we calculated the Pearson correlation coefficient (PCC) among all features, randomly excluding one feature from each pair of features with a correlation coefficient. In accordance with a previous study [11], if the PCC value of the feature pair was > 0.9, we randomly removed one of the features. To avoid the “curse of dimensionality,” we used the least-absolute-shrinkage and selection operator (LASSO) logistic feature selection algorithm to screen the most informative image features.

### Radiomics model construction and evaluation

After feature extraction and selection, we separately trained nine popular classifiers—logistic regression (LR), support vector machine (SVM), decision tree (DT), random forest (RF), AdaBoost (AdaB), gradient boosting (GB), XG boost (XGB), K-nearest neighbors (KNN) and stochastic gradient descent (SGD)—to construct and select an optimal radiomics model for classification of Gram-positive and Gram-negative bacterial pneumonias and atypical bacterial pneumonia. The parameters of model were determined with a grid search technique, which calculates different combinations of model parameter values and each combination was tested with fivefold cross-validation. For each parameter combination, the average area under receiver-operating characteristic curve (AUC) of the five validation sets was calculated. Then the best value was considered as the final choice of the parameters. Finally, we retrain the model on the whole data except the test set based on the best parameters. SGD model tuning parameters are as follows, ‘loss’, ‘penalty’ and ‘alpha’. About Optimal parameters for SGD, which was used with linear support vector machine algorithm, the classification loss function was hinge. The regularization method of the stochastic gradient descent was L2. The alpha was 0.0001, with a data shuffling after each iteration. We also calculated accuracy (ACC), sensitivity (SEN) and specificity (SPE).

### Statistical analysis

Before modeling, we assessed differences in clinical factors among the Gram-positive bacterial pneumonia, Gram-negative bacterial pneumonia and atypical bacterial pneumonia groups. The Student’s *t* test or Kruskal–Wallis *H* test was used for continuous variables, the *χ*^2^ test or Fisher’s exact test for categorical variables. We analyzed all data using SPSS for Windows version 20.0 (IBM Corp., Armonk, New York, US). *P* < 0.05 was considered statistically significant.

## Results

### Patient characteristics

A total of 389 children were included in this study, 122 with Gram-positive bacterial pneumonia, 159 with Gram-negative bacterial pneumonia and 108 with atypical bacterial pneumonia. Patient characteristics in the training and test sets are listed in Table 1. No significant differences were observed between the two sets in age (*P* = 0.370), sex (*P* = 0.053) or other clinical characteristics (*P* > 0.05). A total of 334 patients used antibiotics before admission, 55 did not use before. In addition, 136 patients used only one kind of antibiotic, 88 used two kinds, 33 used three kinds, 6 used more than three kinds of antibiotics and 77 patients cannot explain the kind of antibiotics. All patients have clinical pneumonia but cannot identify the bacteria and no cases have been examined for immunodeficiency. There was a statistically significant difference in age among Gram-positive bacterial pneumonia, Gram-negative bacterial pneumonia and atypical bacterial pneumonia in both the training and test sets (*P* < 0.05). On average, children with atypical bacterial pneumonia were the oldest, those with Gram-positive bacterial pneumonia were younger and those with Gram-negative bacterial pneumonia were youngest.

**Table 1:** Clinical characteristics of children in the training and test sets (*n* = 389)

Variable	Training set				Test set				*p*
	Gram-positive bacteria	Gram-negative bacteria	Atypical bacteria	*p*	Gram-positive bacteria	Gram-negative bacteria	Atypical bacteria	*p*	
Age (year, mean ± SD)	2.20 ± 2.87	1.32 ± 1.91	4.43 ± 3.58	0.000	1.94 ± 2.54	0.85 ± 0.82	4.27 ± 3.85	0.002	0.370
Sex *n* (%)									0.053
Boy	54 (28.0%)	90 (46.6%)	49 (25.4%)	0.000	20 (33.9%)	27 (45.8%)	12 (20.3%)	0.057	
Girl	43 (36.8%)	37 (31.6%)	37 (31.6%)	0.735	5 (25.0%)	5 (25.0%)	10 (50.0%)	0.287	
Fever (*n*)	41 (42.2%)	51 (40.1%)	56 (65.1%)	0.307	12 (30.8%)	15 (38.5%)	12 (30.8%)	0.794	0.895
Cough (*n*)	86 (88.6%)	122 (96.0%)	81 (94.1)	0.326	23 (30.3%)	31 (40.8%)	22 (28.9%)	0.383	0.472
Other symptoms (*n*)	31 (31.9%)	50 (39.2)	25 (29.0)	0.008	11 (30.6%)	16 (44.4%)	9 (25.0%)	0.339	0.081
Course time (day)	12.86 ± 12.09	13.84 ± 14.00	10.10 ± 8.24	0.143	10.44 ± 7.95	12.50 ± 14.57	14.41 ± 12.40	0.184	0.875
Length of stay (day)	9.76 ± 5.82	9.57 ± 5.45	10.30 ± 5.39	0.363	8.24 ± 4.93	9.50 ± 4.80	8.59 ± 3.45	0.785	0.079
Use of antibiotics before admission	79 (29.4%)	108 (40.1%)	82 (30.5%)	0.473	18 (27.7%)	26 (40.0%)	21 (32.3%)	0.471	0.399
With congenital cardiovascular diseases	14 (50%)	12 (42.9%)	2 (7.1%)	0.012	2 (50.0%)	2 (50.0%)	0(0.0%)	0.617	0.359
With congenital blood system diseases	4 (28.6%)	7 (50.0%)	3 (21.4%)	0.395	2 (50.0%)	2 (50.0%)	0 (0.0%)	0.617	0.926
Severe pneumonia	24 (32.9%)	29 (39.7%)	20 (27.4%)	0.434	10 (45.5%)	9 (40.9%)	3 (13.6%)	0.142	0.517

Other symptoms including wheeze, muscle aches, headache, nausea, diarrhea, abdominal pain, shortness of breath and vomiting

*Yr* year; *std* standard deviation. Independent *t *test or Kruskal–Wallis *H* test for continuous variables and the Chi-square test or Fisher’s exact test for categorical variables. A *p* value < 0.05 was considered a statistically significant difference

### Radiomics feature extraction and selection

Before image segmentation, we determined auto-segmentation accuracy. Next, we analyzed our pneumonia segmentation model using low-dose CT images from 30 patients randomly selected from the entire data set. The Dice coefficient (DC) was chosen as the evaluation metric. In these 30 patients, average DC value was 0.835 ± 0.037, suggesting a good segmentation result.

**Fig. 5:**
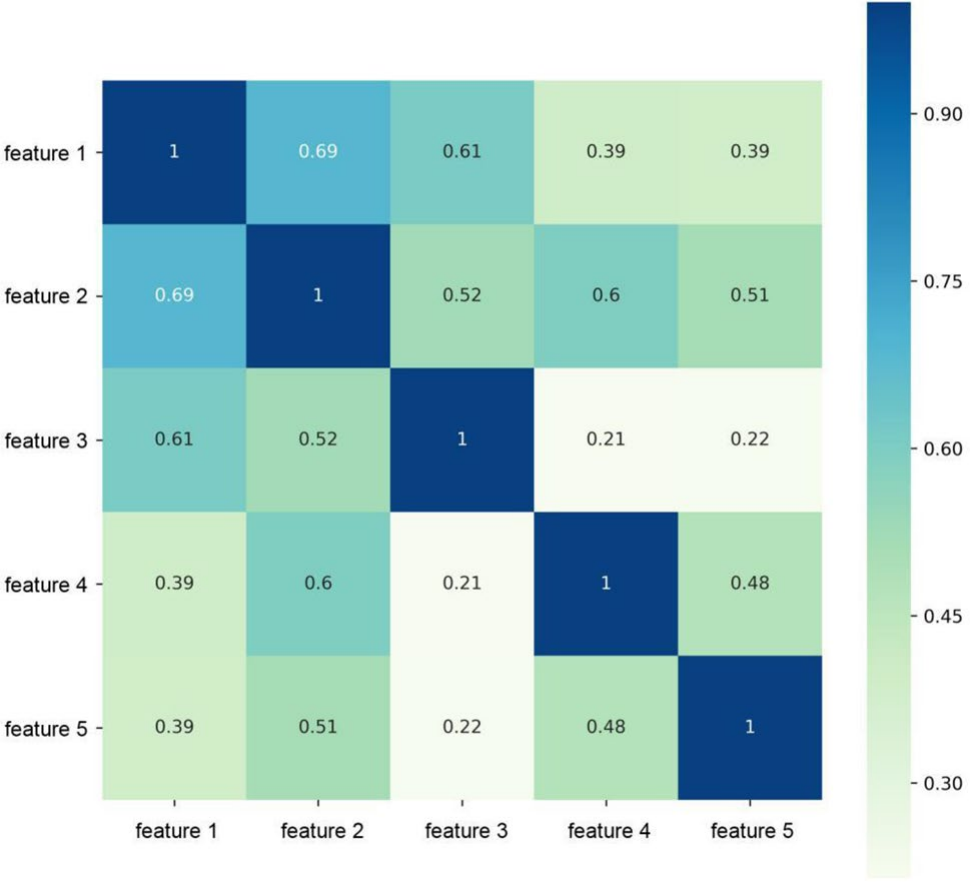
Heat maps of the correlation of five features selected to construct radiomics model. Feature 1, 2, 3, 4, 5 are Original_glszm_SizeZoneNonUniformity, Log-sigma-1–0-mm-3D_firstorder_Maximum,Wavelet-HHH_gldm_LargeDependenceHighGrayLevelEmphasis, Original_glszm_GrayLevelVariance, Wavelet-LLL_firstorder_RootMeanSquared, respectively

In the intra-reader class, 1154 of 1218 (94.7%) radiomics features had good agreement with intra-class coefficients (range 0.750–0.999). In the inter-reader class, 1152 of 1218 (94.5%) radiomics features had good agreement with inter-reader class coefficients (range 0.751–0.999). After PCC filtering, the number of features dropped from 1144 to 220. Based on LASSO dimension reduction, when the penalty parameter of *λ* was 0.008, the error of classification value was lowest. We selected five features related to patient pneumonia type to construct ML models. Heatmaps correlating these five features to construct a radiomics model are shown in Fig. 5.

### Construction and validation of the radiomics model

The development of our comprehensive multi-class classification model was based on the construction of three radiomics submodels: Gram-positive bacterial pneumonia plus the other two types of bacterial pneumonia, Gram-negative bacterial pneumonia plus the other two types and atypical bacterial pneumonia plus the other two types. We used four evaluation indicators—AUC, SEN, SPE and ACC—to assess how well the radiomics model diagnosed and evaluated bacterial pneumonia in the training and test sets. Table 2 presents a comprehensive prediction performance comparison of each classifier in the test set. Pairwise comparisons of the ROC curves were conducted using the DeLong test method. Of the four evaluation indicators, SVM had the best performance, and its AUC was significantly higher than the AUCs of the AdaB, DT and SGD models (*P* < 0.05).

**Table 2:** The performance of nine popular classifiers in test set

	LR	SVM	DT	RF	AdaB	GB	XGB	KNN	SGD
AUC (95% CI)	0.68 (0.56, 0.75)	0.73 (0.61, 0.79)	0.59 (0.50, 0.69)	0.68 (0.55, 0.72)	0.63 (0.54, 0.71)	0.66 (0.58,0.76)	0.65 (0.59, 0.74)	0.65 (0.54,0.71)	0.6 (0.55.0.71)
ACC	0.53	0.54	0.47	0.5	0.51	0.5	0.47	0.5	0.43
SEN	0.51	0.52	0.46	0.46	0.46	0.48	0.45	0.47	0.43
SPE	0.52	0.75	0.73	0.74	0.73	0.73	0.72	0.73	0.71

*LR* logistic regression, *SVM* support vector machine, *DT* decision tree, *RF* random forest, *AdaB* AdaBoost, *GB* gradient boosting, *XGB* XG boost, *KNN* K-nearest neighbors, *SGD* stochastic gradient descent, *ROC* receiver-operating characteristic, *AUC* area under the curve, *ACC* accuracy, *SEN* sensitivity, *SPE* specificity

**Fig. 6:**
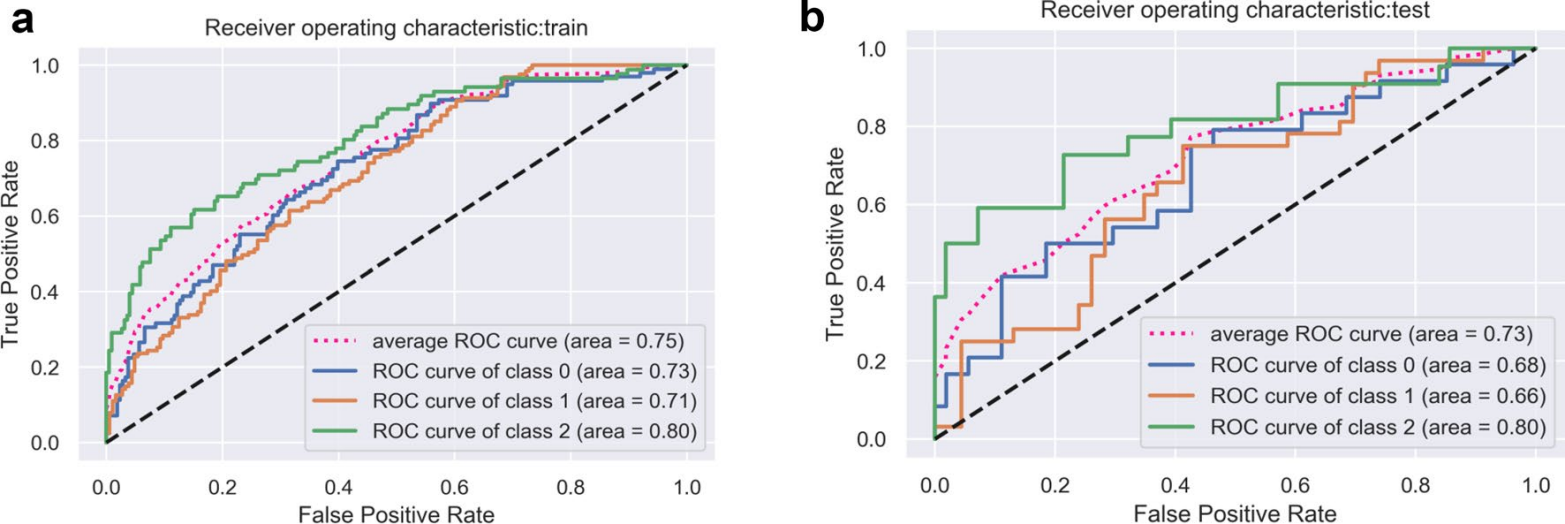
Receiver-operating characteristic (ROC) curves in the training set (**a**) and test set (**b**)

**Fig. 7:**
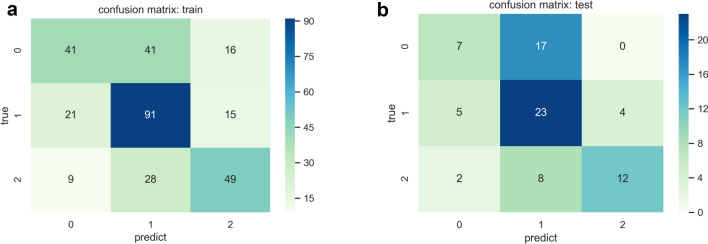
Confusion matrix diagram in the training set (**a**) and test set (**b**)

For comparison, we input each group of features to optimize the regression model to classify the three pneumonia types. Their respective and fused predictive efficacies are shown in Table 3. AUC value range was 0.59–0.75, indicating that each group of features had significantly higher classified value in this task than random guess, which had an AUC of 0.3. Ultimately, we chose the SVM classifier to construct our comprehensive radiomics model. In general, this model achieved satisfactory performance, with an average AUC of 0.75 (95% CI 0.65–0.83) and ACC of 0.58 (SEN, 0.57; SPE, 0.78) in the training set and an average AUC of 0.73 (95% CI 0.61–0.79) and ACC of 0.54 (SEN, 0.52; SPE, 0.75) in the test set (Figs. 6, 7; Table 4).

**Table 3:** Classified efficacy of five radiomic features in test set

Feature type	AUC
Fused feature(SVM)	0.73
Fused feature(LR)	0.68
original_glszm_GrayLevelVariance	0.64
original_glszm_SizeZoneNonUniformity	0.63
log-sigma-1–0-mm-3D_firstorder_Maximum	0.61
wavelet-LLL_firstorder_RootMeanSquared	0.61
wavelet-HHH_gldm_LargeDependenceHighGrayLevelEmphasis	0.55

**Table 4:** Performance of SVM model in training and test set

	Models	Total patients	Positive patients	AUC	AUC(95% CI)	ACC	SEN	SPE
Training set	Gram-positive bacterial pneumonia model	311	98	0.72	(0.63,0.81)	0.72	0.42	0.86
	Gram-negative bacterial pneumonia	311	127	0.71	(0.61,0.80)	0.66	0.72	0.63
	Atypical bacterial pneumonia	311	86	0.80	(0.72,0.88)	0.78	0.57	0.86
	Average	311	311	0.75	(0.65,0.83)	0.58	0.57	0.78
Test set	Gram-positive bacterial pneumonia	78	24	0.68	(0.58,0.75)	0.69	0.29	0.87
	Gram-negative bacterial pneumonia	78	32	0.66	(0.58,0.76)	0.56	0.72	0.46
	Atypical bacterial pneumonia	78	22	0.80	(0.67,0.85)	0.82	0.55	0.93
	Average	78	78	0.73	(0.61,0.79)	0.54	0.52	0.75

Positive patients refers to the number of patients with each group of bacterial pneumonia

*AUC *area under the curve, *ACC *accuracy, *SEN *sensitivity, *SPE *specificity

## Discussion

In this study, we separately found five different radiomics features related to the multi-class classification of Gram-positive, Gram-negative and atypical bacterial pneumonias. We then constructed and validated a comprehensive multi-class classification model for differential diagnosis of these three common types of bacterial pneumonia by synthesizing three radiomics submodels based on radiomics features extracted from low-dose chest CT images. The results of our study indicated that the atypical pneumonia model performed better than either the Gram-positive or Gram-negative model. Compared with traditional imaging, in which radiologists can rarely distinguish type of bacterial pneumonia by reading CT images only, the radiomics model could provide a reference for continual optimization in the future. Note that our current results are not ideal, which might be related to the instinct characteristic of multi-classification method and the pathological manifestations of bacterial pneumonia.

Considering the child’s cooperation and radiation damage, a chest X-ray is the optimal option for diagnosing pneumonia. But CT needs to be considered when complications are suspected or there is difficulty in differentiating community-acquired pneumonia from other pathology [12]. We should exclude other reasons beside infections causing pneumonia such as obstruction and tumor and so on. Over the past decade, recent advances in CT scanning technology have dramatically reduced radiation doses [13]. The radiation dose of a low-dose chest CT is within 1 mSv; however, compared with 0.1 mSv for a chest X-ray, the radiation dose is still important to consider for the application of CT in children [14]. However, X-ray might not be the ideal way to identify the cause of pneumonia. X-ray interpretation has high inter-observer variability, particularly between unexperienced and experienced readers [15], leading to both underdiagnosis and overdiagnosis. Withholding antibiotics can cause the physician to miss the best time to treat pneumonia and prolong the course of the disease, while the opposite error can lead to antibiotic resistance. Therefore, correct diagnosis and avoidance of antibiotic overuse should be given priority over radiation dose and economic costs [12]. Accordingly, we chose low-dose chest CT for early diagnosis of pediatric pneumonia. Owing to its intrinsic advantages and better results than other machine-learning (ML) models, we chose SVM classifier. SVM achieved good performance when classifying image data, even with limited clinical samples. We used L2-term regularization to improve generalization capabilities and prevent overfitting, as well as the kernel trick to handle nonlinear data to improve their flexibility and adaptability. We defined the range of values for each hyperparameter; for example, *C* = {0.01, 0.1, 1.0, 10, 100}. The grid search algorithm was guided by the AUC measured on the training set. Optimal *C* and gamma were both 1.0.

Many clinical manifestations of bacterial infection, as well as extrapulmonary complications, are due to immunopathological and inflammatory effects produced by the host rather than by the organism itself. Different bacterial infections can produce similar immune responses and symptoms. Therefore, we assumed that radiomics features were related to the pathogenic mechanism of bacterial infection. The first two features, gray-level variance (GLV) and size zone non-uniformity (SZN), with the highest correlation in the comprehensive model, both belonged to the GLSZM group. GLV measures the variance in gray-level intensities for the zones. SZN measures the variability of size zone volumes in the image, indicating the degree of homogeneity in these volumes and thus differences in the homogeneity of pulmonary lesions caused by the three types of bacterial infection. Although the infected area, shape or grayscale may be consistent, the microscopic distribution of lesions caused by one type of bacterial infection might be relatively concentrated, while those caused by another might be relatively dispersed.

Though over several years, the specific pathogeneses of Gram-positive, Gram-negative and atypical bacterial pneumonia infections are complex and require further research. The pathogenic mechanism of atypical bacterial pneumonia, including CPP, MPP and CTP, differs too much from those of Gram-positive and Gram-negative bacterial pneumonia infections. For example, *M*. *pneumoniae* is the smallest prokaryotic microbe in nature; its lack of walls and malleability facilitate its adhesion to host tissues. Its membrane proteins, invasive proteins and adhesive proteins enable it to pass through cell filters, causing adhesion damage, membrane fusion damage, nutrition depletion, invasive damage, toxic damage, immune damage and inflammatory damage [16]. This is its main pathogenic mechanism. Therefore, characteristics such as bronchial-wall thickening, lateral bronchial-wall thickening, or intralobular or lobular ground-glass opacities can help us distinguish MPP from other bacterial pneumonias [17].

However, the major pathogenic mechanism of Gram-positive and Gram-negative bacteria upon entering the host environment (e.g., nasal colonization) is to express myriad bacterial-virulence factors that cause bacterial pore-forming toxins (PFTs), host cell death and inflammation activation [18]. The difference between Gram-positive and Gram-negative bacteria is that they release different types of toxins, making it difficult to find significant differences between these two types on CT images. Therefore, Gram-positive and Gram-negative bacterial pneumonias are easier than atypical bacterial pneumonia to identify via radiomics features.

There were several limitations to the present study. First, the dataset was from a single center; however, real-world situations may be different, particularly in terms of disease prevalence and diversity of children. Therefore, further external validation and clinical-utility tests in various clinical settings are warranted. Second, our results were not ideal because this was a multi-class classification model that need more clinical factors or multiple dimensions of indicators be into consideration. Third, the DC value was not so high of the automatic segmentation model that need to be further improved in the future study by improving algorithm or the amount of data.

In conclusion, using an AI-based method, we established a radiomics multi-class classification model for EAT based on low-dose chest CT images of children with Gram-positive bacterial pneumonia, Gram-negative bacterial pneumonia and atypical bacterial pneumonia. This is a very new type of model on which we plan to improve. We hope that in the future, this radiomics model can be applied using a convenient software package that connects to PACS in radiology departments, and that radiologists will recommend EAT to clinicians based on the results of this model when reporting conventional CT findings of pneumonia like making a suspiciously malignant or benign statement when diagnosing a tumor.

## Data Availability

The database used and/or analyzed during the current study are available from the corresponding author on reasonable request.

## Abbreviations

EAT: Empirical antibiotic therapy
CAP: Community-acquired pneumonia
CPP: *Chlamydia pneumoniae* Pneumonia
MPP: *Mycoplasma pneumoniae* Pneumonia
CTP: *Chlamydia trachomatis* Pneumonia
CT: Computed tomography
AI: Artificial intelligence
LASSO: Least-absolute-shrinkage and selection operator
GLCM: Gray-level co-occurrence matrix
GLRLM: Gray-level run length matrix
GLSZM: Gray-level size zone matrix
GLDM: Gray-level dependence matrix
ROI: Region of interest
ML: Machine learning
LR: Logistic regression
SVM: Support vector machine
DT: Decision tree
RF: Random forest
AdaB: AdaBoost
GB: Gradient boosting
XGB: XG boost
KNN: K-nearest neighbors
SGD: Stochastic gradient descent
ROC: Receiver-operating characteristic
AUC: Area under the curve
ACC: Accuracy
SEN: Sensitivity
SPE: Specificity

## Code availability

The code used and/or analyzed during the current study is available from the corresponding author on reasonable request.
